# A systems-wide comparison of red rice (*Oryza longistaminata*) tissues identifies rhizome specific genes and proteins that are targets for cultivated rice improvement

**DOI:** 10.1186/1471-2229-14-46

**Published:** 2014-02-12

**Authors:** Ruifeng He, Fernanda Salvato, Jeong-Jin Park, Min-Jeong Kim, William Nelson, Tiago S Balbuena, Mark Willer, John A Crow, Greg D May, Carol A Soderlund, Jay J Thelen, David R Gang

**Affiliations:** 1 Institute of Biological Chemistry, Washington State University, PO Box 646340, Pullman, WA 99164, USA; 2 Department of Biochemistry and Interdisciplinary Plant Group, University of Missouri, Christopher S. Bond Life Sciences Center, Columbia, MO 65211, USA; 3 BIO5 Institute, The University of Arizona, Tucson, AZ 85721, USA; 4 National Center for Genome Resources, Santa Fe, NM 87505, USA; 5 Current Address: Departamento de Tecnologia, Universidade Estadual Paulista, Jaboticabal, SP 14884-900, Brazil

**Keywords:** Transcriptomics, Proteomics, Metabolomics, Rhizome, Invasive species, Disease resistance, Rice blast, Rice

## Abstract

**Background:**

The rhizome, the original stem of land plants, enables species to invade new territory and is a critical component of perenniality, especially in grasses. Red rice (*Oryza longistaminata*) is a perennial wild rice species with many valuable traits that could be used to improve cultivated rice cultivars, including rhizomatousness, disease resistance and drought tolerance. Despite these features, little is known about the molecular mechanisms that contribute to rhizome growth, development and function in this plant.

**Results:**

We used an integrated approach to compare the transcriptome, proteome and metabolome of the rhizome to other tissues of red rice. 116 Gb of transcriptome sequence was obtained from various tissues and used to identify rhizome-specific and preferentially expressed genes, including transcription factors and hormone metabolism and stress response-related genes. Proteomics and metabolomics approaches identified 41 proteins and more than 100 primary metabolites and plant hormones with rhizome preferential accumulation. Of particular interest was the identification of a large number of gene transcripts from *Magnaportha oryzae*, the fungus that causes rice blast disease in cultivated rice, even though the red rice plants showed no sign of disease.

**Conclusions:**

A significant set of genes, proteins and metabolites appear to be specifically or preferentially expressed in the rhizome of *O. longistaminata*. The presence of *M. oryzae* gene transcripts at a high level in apparently healthy plants suggests that red rice is resistant to this pathogen, and may be able to provide genes to cultivated rice that will enable resistance to rice blast disease.

## Background

Rice is the most economically important crop in the world and is the principal cereal in the human diet, supplying humanity with 23% of its calories, followed by wheat (17%) and maize (10%) [1]. The genus *Oryza* consists of two cultivated species, *O. sativa* (the Asian cultivated rice) and *O. glaberrima* (the African cultivated rice), and more than 20 wild species, many of which are consumed in the human diet in certain regions of the world. The wild species of *Oryza*, representing the AA, BB, CC, BBCC, CCDD, DD(EE), FF, GG, HHJJ and HHKK genomes provide an important pool of useful genes for rice improvement [2,3].

The red rice, *Oryza longistaminata*, is a rhizomatous perennial wild rice species native to Africa and considered a noxious weed in the United States. It is believed to be the wild progenitor of *O. glabberrima*. The genome of *O. longistaminata* is type AA, similar to *O. sativa*. This species is characterized by a number of valuable traits that could be used for improvement of rice cultivars, including long anther [4], large biomass [5], high nitrogen use efficiency [6], resistance to insect pests and disease [7], and the rhizomatous trait. Elucidation of the genetic origin of the rhizomatous trait might facilitate the generation of a perennial form of cultivated rice that would help to reduce soil erosion associated with annual tillage.

Rhizomes are essential organs to many perennial species by enabling overwintering and allowing rapid growth at the beginning of the next growing season [8]. Plants regrowing from rhizomes have the potential for greater vigor and faster early growth than plants grown in a traditional ratoon system [9]. *O. longistaminata* is the most logical donor of perenniality for cultivated rice because of its strong rhizome features and relatively high pollen fertility compared to other wild rice species. However, hybrid sterility is currently a considerable barrier to rapid development of *O. longistaminata/O. sativa* crosses [10,11].

A cross between cultivated sorghum and the perennial grass, *Sorghum propinquum*, was made in an effort to map genes for rhizome development [12]. Some genes with rhizome-enriched expression were identified in the *Sorghum* rhizome species Johnsongrass (*S. halepense*, 2n = 2x = 40) and *S. propinquum* (2n = 2x = 20) [13,14], in bamboo (*Phyllostachys praecox*) [15], and especially in red rice (*O. longistaminata*) [16,17]. The combined results from those investigations indicated that a complex gene regulatory network may underlie rhizome development and growth.

Despite the significance of rhizomes in red rice, little is known about the molecular basis of rhizome growth, development, and function in this species. To broaden our understanding of the global gene expression profiles of *O. longistaminata* rhizomes and to identify the genes and proteins involved in rhizome development and function, we utilized the next-generation high-throughput sequencing platforms Illumina GAII and HiSeq 2000 to sequence the rhizome transcriptome and to compare gene expression profiles of rhizomes with other tissues, respectively. In parallel, we employed label-free quantitative proteomics that utilized gel electrophoresis and liquid chromatography-tandem mass spectrometry (GeLC-MS/MS) to globally query the proteome of rhizome and root tissues. We also investigated primary metabolite and hormone levels in the rhizome and other plant tissues. We then compared the transcriptomic, proteomic, and metabolomic results to investigate rhizome specificity and identify genes, proteins and metabolites that appear to play important roles in rhizome development and function. The data and results can be viewed at http://www.plantrhizome.org.

## Results and discussion

### Transcriptomics and differential expression analysis

**Figure 1:**
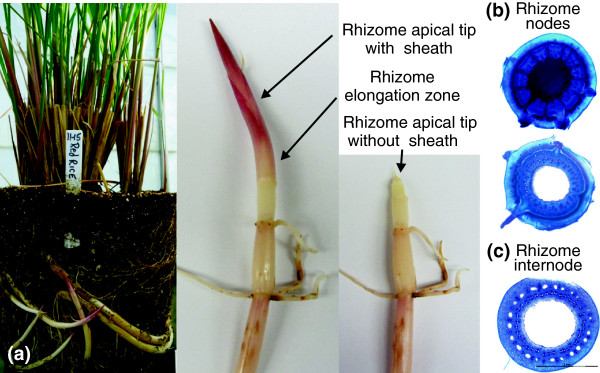
**Red rice (*Oryza longistaminata *) plant and rhizome. (a)** Plants (left) with strong rhizomes grow well in the greenhouse. The rhizome apical tips and the elongation zones of the rhizomes (right) were harvested for RNA, protein and metabolite isolation. **(b)** The anatomy of a cross section of the rhizome node. **(c)** A cross section of the rhizome internode.

We collected rhizome apical tips (containing the meristematic region, “tip” for short), the neighboring elongation zones (“zone” for short), whole rhizome (including rhizome tip and zone) and other tissue samples (root, stem and leaf) from *O. longistaminata* plants at the active tillering stage (Figure 1). The total transcriptomes from rhizome elongation zone and apical tip tissues, as well as RNA-seq data from other tissues, were obtained using Illumina-based next-generation sequencing technology (see Methods), yielding more than 465 million 100 bp reads from the rhizome tip and 450 million from the rhizome zone, as well as 135, 129, 151 and 143 million 50 bp reads from whole rhizome, root, leaf and stem tissue samples, respectively. In total, we obtained 116 Gb of transcriptome data with more than 1,476 million cleaned reads, of which 1,284 million reads were assembled as described in the Methods section to generate 143,625 unigenes (assembled cDNA sequences). The assembled transcriptomic data amount was 101 Mb (about one fourth of the genome size) with an average GC content of 45%, ranging from 11 to 84% per unigene (Table 1).

**Table 1:** Summary of red rice transcriptome data

Total reads (million)	1,476
Total nucleotide length (Gb)	116
Total unigenes	143,625
Average expression of unigenes (RPKM)	32
Mean length of unigenes (range) (bp)	703 (200- 16,522)
Assembled data amount (Mb)	101
% GC (range)	45 (11- 84)
Total annotated unigenes (%)	56,232 (39%)
Unigenes with GOs	45,058 (31%)
Protein ORF >100	33,696 (23%)

Note: Unigenes- the consensus transcript sequences; RPKM - reads per KB per million reads; GO - gene ontology; ORF - open reading frame.

Of the 143,625 total unigenes from the “OlR” (*O. longistaminata* Rhizome project) database, 56,232 unigenes (39%) were annotated as matching a known gene in the UniProt or PlantTFDB databases. While the majority of the 56,232 annotated unigenes were homologous to genes from plants (46,454 or 83%), 5,985 (11%) unigenes matched fungal genes, which were mainly assigned to the rhizome tissues (see below) (Table 2) and 1,923 (4%) matched invertebrate, vertebrate and viral genes.

**Table 2:** Fungal genes in red rice rhizome tissues

	**Number**	**Average expression levels (RPKM)**				
		**Leaf**	**Stem**	**Root**	**Rhizome tip**	**Rhizome zone**
Annotated as plant genes	46,454 (83%)	13	12.5	12.8	12.8	12.8
Annotated as fungal genes	5,985 (11%)	0.06	0.13	0.31	0.33	0.82

**Figure 2:**
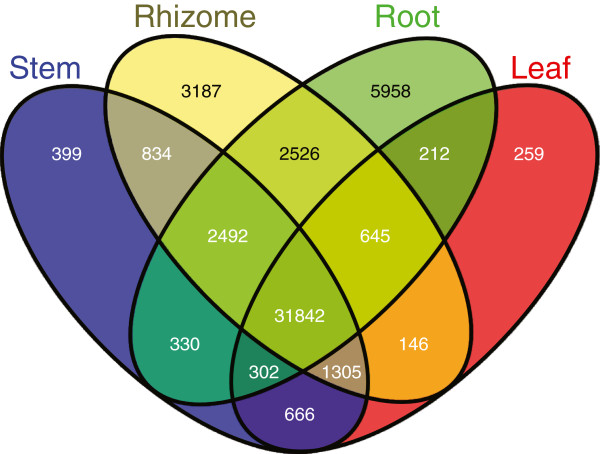
**Venn diagram showing unigene distribution among different tissues.** A total of 3,187 unigenes were from rhizome only. A total of 31,842 unigenes were common to all four tissues: rhizome, root, stem and leaf.

The majority of the annotated genes had expression levels of at least 0.1 RPKM. This included 42,977genes expressed in any rhizome tissue, 36,231 found in all rhizome tissues, and 44,307 expressed in root, 38,170 in stem, and 35,377 in leaf. A small fraction of the genes were specifically expressed in only one tissue, having an RPKM value of at least 0.1 in one tissue but zero from all other tissues: 3,187 unigenes from any rhizome tissue, 5,958 only in root, 399 in stem, and 259 in leaf. A total of 31,842 unigenes were common to all tissues using an expression level of 0.1 RPKM as a cutoff value (Figure 2).

5,985 fungal unigenes were assigned to 103 fungal species with the highest number of unigenes (3705, 62%) from *Magnaporthe oryzae*. In the list of top 20 most abundantly expressed fungal unigenes (Additional file 1), five unigenes were from *Magnaporthe oryzae* and the rest of them (15 unigenes) were from 12 different fungal species. Most of these unigenes had higher expression levels in rhizome and root than in leaf and stem. One unigene, OlR_137032, had 20-fold higher expression in rhizome than in root, but another unigene, OlR_121956, had 20-fold high expression in root than in rhizome. The single most abundant unigene (OlR_051552) was assigned to fungal species *Ustilago maydis* with high expression levels in all tissues (366 RPKM in leaf, 197 RPKM in stem, 631 RPKM in root and 531 RPKM in rhizome).

Interestingly, 1,831 (57%) of the 3,187 unigenes expressed specifically in the rhizome were homologous to fungal genes, with 1,184 (37%) being from *M. oryzae,* a hemibiotrophic fungal pathogen that causes rice blast. The remaining rhizome-specific genes included genes assigned to plants (37%), invertebrates (5%), vertebrates (1%) and other fungi besides *M. oryzae* (20%). Among the remaining 20% fungal genes, most were homologous to *Colletotrichum* genes with the highest relative abundance (6%, 100 unigenes). *Thielavia* (4%, 69 unigenes) was the second most abundant group, followed by *Neurospora* (66 unigenes), *Chaetomium* (60 unigenes), *Hypocrea/Trichoderma* (43 unigenes), *Podospora* (43 unigenes), *Grosmannia* (30 unigenes), *Nectria* (30 unigenes), *Fusarium* (25 unigenes), *Metarhizium* (17 unigenes) and *Aspergillus* (16 unigenes), etc. (see Additional file 2).

Some of these fungal species are resistant to abiotic stress such as heat or extreme environments. For example, *Thielavia* is a thermophilic fungus, and may play roles in biomass conversion [18]. *Chaetomium thermophilum* is also thermophilic. The unigenes of thermophilic fungi had relatively higher expression levels in rhizome tissues than other tissues (Additional file 2). Some fungi are endophytic, such as *Chaetomium globosum*. These endophytic fungi produce antifungal metabolites and were used for biocontrol of rice blast [19], wheat leaf rust [19], potato late blight [20] and corn leaf blight [21]. The species of *Hypocrea/Trichoderma* are also well studied as biocontrol agents with the ability to interact with plant roots/rhizomes and act as plant symbionts to generate cell wall hydrolytic enzymes and antagonistic secondary metabolites to compete against soil-borne pathogens [22,23]. Members of the genus *Metarhizium* include fungi pathogenic to insects, produce insecticidally active compounds [24] and were used to develop biocontrol alternatives to chemical insecticides in agricultural and disease-vector control programs [25]. Although the genera *Podospora*, *Grosmannia*, *Nectria*, *Fusarium* and *Colletotrichum* contain plant pathogens and cause serious plant disease, some of them such as *Colletotrichum* species are also frequently identified as endophytes, which are commonly isolated from healthy plants, including medicinal plants and rhizome species [26,27]. *M. oryzae* acts as a pathogen to numerous plant species, especially to cultivated rice, but it may be present as an endophyte in red rice. It is dominant in the rhizome tissue and may function as either a beneficial endophyte or pathogen depending on the circumstances and plant hosts.

The fact that the genes from thermophilic fungi, entomopathogenic fungi and endophytic fungi were dominant in the rhizome tissues and some of these showed high expression levels indicated that these fungal genes may play important roles in rhizome tissue. There is increasing evidence that many fungi behave as endophytes in plants, especially in grasses [28,29], and they may be involved in plant defense against pathogens and insect pests [30]. Moreover, fungal endosymbionts may play roles in conferring plant abiotic stress tolerance [31] or may affect the biosynthesis and accumulation of bioactive metabolites [30,32]. In recent years, endophytes, especially those from medicinal plants, rhizome species and invasive species have become the focus of research for identification of bioactive compounds [26,33], for invasive plant management and plant community biodiversity [34-37].

On the other hand, just 227 of 5,958 root-specific annotated unigenes appeared to be fungal genes with 13 from *M. oryzae* and four from *Colletotrichum*. The others included *Aspergillus* (19 unigenes), *Chaetomium* (13 unigenes), *Hypocrea/Trichoderma* (11 unigenes), *Thielavia* (8 unigenes), etc. Interestingly, there were 12 unigenes that were homologus to *Coprinopsis cinerea*, and some of these genes showed high expression levels, but only one unigene from *Coprinopsis cinerea* was found to be specifically expressed in the rhizome. In addition, a unigene, OlR_050405, encoding a cellobiohydrolase from *Irpex lacteus* showed very high expression levels (64 RPKM) and was specifically expressed in the root tissues (Additional file 3). An ornithine decarboxylase gene assigned to *Glomus intraradices* was also detected in root. *Glomus intraradices* is an arbuscular mycorrhizal (AM) fungus used as a soil inoculant in agriculture and horticulture. These results indicated that different fungal species exist in root and rhizome tissues of red rice and that they express some genes to levels that exceeded those of the host plant. Notably, rhizome tissues supported a more abundant and diverse fungal population, and appeared to particularly host *Magnaporthe oryzae*.

**Figure 3:**
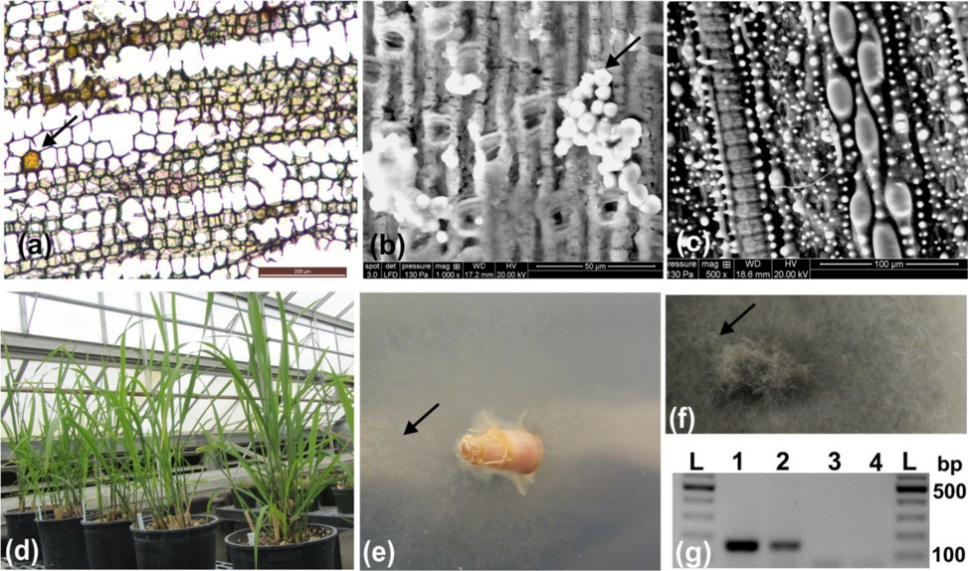
**Cytological and molecular analysis of *M. oryzae *in rhizome tissues. (a)** Rhizome tissue sections were stained by Toluidine blue and visualized by light microscopy. Brown deposition/Rice blast lesions in individual epidermal cells were observed (arrow) but did not show spreading of the lesions on the rhizome tissue surface. Bar = 200 μm. **(b)** Fungal conidia were observed on the surface of rhizome (arrow) by scanning electron micrograph (SEM). Bar = 50 μm. **(c)** No fungal conidia or infection hyphae on red rice leaf tissues were detected. Bar = 100 μm. **(d)** The red rice plants grew in the greenhouse without any typical symptoms of rice blast/*M. oryzae*. **(e)** Fungal hyphae (arrow) spread on the Petri dish from the rhizome tissue with three days of culture on potao dextrose agar (PDA). **(f)** Fungal hyphae and spores (arrow) grew on the Petri dish from the rhizome tissue with eight days of culture on PDA. **(g)** An *M. oryzae-*specific gene was detected by RT-PCR, showing amplification of the predicted specific gene sequence 156 bp fragment in hyphyae/mycelia (lane 1) and rhizome (lane 2), but not in root (lane 3) and leaf (lane 4). L: DNA ladder.

Among the 5,985 unigenes that were derived from fungal endophytes/symbionts or pathogens/contaminants, the average expression levels (RPKM) were 0.06 in leaf, 0.13 in stem, 0.31 in root, 0.33 in rhizome tip and 0.82 in rhizome elongation zone (Table 2). In comparison, the RPKM values of plant genes were much more consistent and higher, with an average RPKM of 13.0 in leaf, 12.5 in stem, 12.8 in root, and 12.8 in both tip and zone (Table 2). These results indicated that the fungal-related genes were more highly expressed in the rhizome tissue, especially in the rhizome elongation zone, compared to other tissues. For example, the unigene (OlR_100675) assigned to alcohol oxidase of *M. oryzae* was specifically expressed in the rhizome tissue (Additional file 2). This gene was reported to be highly expressed both in appressoria and conidia/mycelia of *M. oryzae*[38], and is a pathogenicity factor gene that was induced by plant-derived signals and carbon starvation, but repressed by nitrogen starvation [39]. Furthermore, using specific primers of the *M. oryzae* alcohol oxidase gene, we detected the alcohol oxidase gene in conidia/mycelia that was induced and purified from healthy rhizome tissues (Figure 3g), indicating that *M. oryzae* was present in red rice rhizomes.

The identification of so many *M. oryzae* genes within red rice was a bit surprising, as the plants used in this investigation did not show symptoms of rice blast disease (Figure 3d). Indeed, none of the red rice plants grown over the past several years in our greenhouses have shown symptoms of rice blast. However, cytological and molecular analysis shows that conidia and hyphae of *M. oryzae* are indeed present in the healthy rhizome tissue (Figure 3). Taken together, the cytological, transcriptomic and proteomic data (see below) indicated that red rice (*O. longistaminata*), as has been found for other wild rice species and some cultivated land races, may be resistant to *M. oryzae* and may even harbor the fungus as an endophyte in a non-pathogenic relationship within the rhizome. Being a potential carrier of rice blast, red rice thus must possess means to control spread within the plant of *M. oryzae* (the transcriptome data outlined herein suggest that this is the case), and may have genes that could be transferred to cultivated rice to endow the latter with the same resistance characteristics. Identification of such genes will be a critical component of future comparative genomics-based investigations of these two species. In the case of *O. rhizomatis*, another rhizomatous wild rice species (but of limited distribution, to Sri Lanka), a unique NBS-LRR gene containing a zinc finger domain has been identified, termed Pi54rh, which may confer resistance to rice blast and is significantly upregulated in response to infection initiation [40]. A close homolog of that gene (~91% identical at the nucleotide level) and represented by unigene OlR_092835 in our dataset was expressed in all red rice tissues examined and may be partially responsible for resistance in red rice. However, it was only slightly upregulated in the rhizome than in other parts of the plant, and thus does not correlate well with the large fraction of *M. oryzae* transcripts found in the rhizome.

In addition to rhizome-specific genes, we were interested in identifying genes that were preferentially expressed in the rhizome compared to other tissues. The sequence data from the three rhizome tissues were combined and compared to the non-rhizome tissues using edgeR [41]. At a p-value < 0.01, there were 19,259 DE (differentially expressed/differential expression, i.e., significantly changed) unigenes compared to root, 18,009 compared to stem, and 21,555 compared to leaf. There were 5,539 DE unigenes between the rhizome tissues and all non-rhizome tissues (i.e., combined rhizome compared to root and stem and leaf). Breaking this down, there were 1,013 unigenes differentially expressed between the apical tip and the non-rhizome tissues, 1,265 DE unigenes between the zone and the non-rhizome tissues, and 7,582 DE unigenes between whole rhizome and the non-rhizome tissues.

The results above that outline the detection of fungal gene in the rhizomes is supported by the following DE results. The OlR database has 372 k unique hits to plants and 155 k unique hits to fungus, of which 3,522 have the best e-value hit to fungus and 1,499 of those were to *M. oryzae*. Of the 5,539 rhizome DE unigenes, 125 had their best hit to fungus, of which 55 were from *M. oryzae*. In contrast, root has 6,958 DE genes compared with all other libraries, where 98 of those were of fungal origin and 4 were from *M. oryzae*. Thus, *M. oryzae* appeared to be localized mostly to the rhizome, based on gene expression data.

Differential expression analysis was also applied to GO categories using GOseq [42], comparing combined rhizome tissues to root, stem, and leaf. The top two biological process categories identified in that analysis were “response to stimulus” and “multi-organism process”. The top three cellular component categories were “extracellular region”, “membrane” and “cell junction”. The top molecular functions were “antioxidant activity” and “electron carrier activity”. These data were used to focus efforts to identify specific genes that may play important roles in determining the growth, development, differentiation and function of the rhizomes of red rice, as outlined below.

### Rhizome-enriched genes

**Figure 4:**
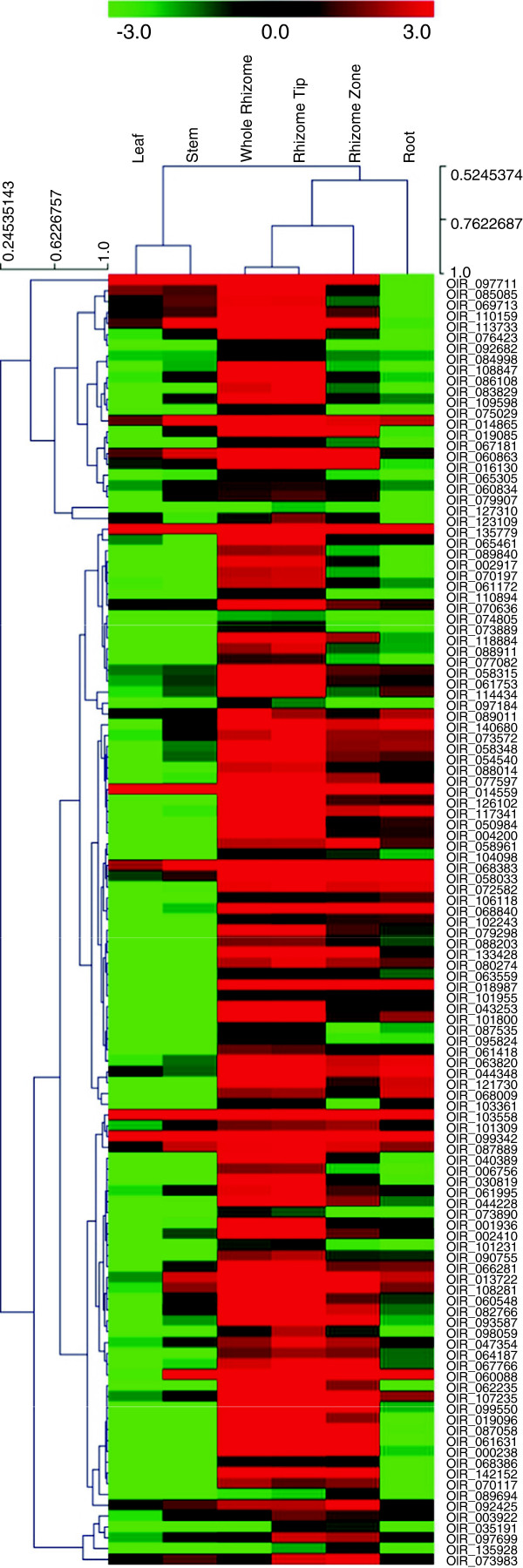
Hierarchical clustering for the 119 rhizome-enriched genes.

Among the genes identified as being differentially expressed in rhizome compared to other tissues (root, stem and leaf), 3,795 displayed > 2-fold higher expression levels in the rhizome. We further identified 119 unigenes/genes that were specifically expressed or highly enriched in the rhizome (especially in the rhizome apical tip) (Figure 4), 40% of which (48 out of 119) were related to known transcription factors, including YABBY, TALE, AP2 and bHLH etc. (Additional file 4). This result was consistent with the previous report in which 58 rhizome tip-specifically expressed genes were identified (based on microarray hybridization), including 15 transcription regulation related genes [17]. Other classes of genes that would be expected to play important roles in rhizome growth and development were also identified, some with preferential expression in either the apical tip or the elongation zone.

Importantly, some classes of phytohormone (e.g., auxin and gibberellin) and stress response related genes likely play key roles in the regulation of rhizome development and environmental responses [43]. Indeed, in the red rice transcriptome dataset, we found 144 phytohormone-related unigenes/genes, including genes related to the production, sensing and metabolism of auxin (78 unigenes), cytokinins (24 unigenes), ethylene (18 unigenes), brassinosteroids (BRs, 11 unigenes), gibberellins (GAs, 8 unigenes) and abscisic acid (ABA, 5 unigenes) (Additional file 5). In particular, up-regulated genes in the rhizome included those encoding gibberellin stimulated transcript related protein 2 (OlR_126102), ethylene receptor-like protein 2 (OlR_035191 and OlR_111522), auxin-responsive protein IAA14 (OlR_061172) and auxin response factor 11 (OlR_065461). Other putative phytohormone-related genes were down-regulated in rhizome relative to the leaf, stem and root, such as an auxin-responsive protein, IAA8 (OlR_133718 and OlR_078134), auxin-induced protein-like protein (OlR_069872 and OlR_015268) and cytokinin dehydrogenase 10 (OlR_028085 and OlR_118428). Similar results were also observed in the previous study [17] that compared isolated RNAs from *O. longistaminata* to the *O. sativa* genome via use of Affymetrix chip hybridization. However, because these genes were sequenced de novo in our investigation, the possibility of cross or non-specific hybridization that could have occurred in the previous study was eliminated, providing stronger evidence for involvement of these genes in rhizome-specific processes. Thus, these genes may play essential roles in the regulation of rhizome development and environmental responses in red rice.

In addition, expansin family genes, growth-regulating factor genes and other stress-related genes such as β-glucosidases, heat shock genes, drought-induced S-like ribonuclease genes, disease resistance genes, an LRR receptor-like serine/threonine-protein kinase and an NB-ARC domain containing protein appear to be preferentially expressed in the rhizome tissue. For example, Expansin-A2 may function in loosening and extension of plant cell walls and may be involved in elongation of internodes during submergence of rice [44], or in red rice in this case. β-Glucosidases have been implicated in physiologically important processes in plants, such as response to biotic and abiotic stresses, and activation of phytohormones and chemical defense compounds [45]. The exact role that these enzymes play in red rice rhizome is yet to be determined. The LRR receptor-like protein kinase gene was highly expressed in other plant species in regions undergoing cell division and elongation and was induced by gibberellins [46]. Most receptor-like kinases (RLKs) identified as being involved in plant defense are of the LRR-RLK class, including the rice Xa21 protein [47]. The NB-ARC (nucleotide-binding adaptor shared by APAF-1, R proteins, and CED-4) domain is usually associated with Resistance (R) proteins in plants, conferring specificity to the innate immune system [48]. Further characterization of these genes/proteins may reveal roles in invasiveness, underground defense and rhizome development in red rice.

Of specific interest with regards to rhizome growth and development are 27 genes that were the most highly and specifically expressed in the rhizome compared to other tissues (Table 3). Of these, 8 are transcription factors, belonging to YABBY, Bzip, TALE and bHLH gene families. Others included a sesquiterpene synthase, proline-rich proteins, a papain-like cysteine proteinase and an *O*-methyltransferase.

**Table 3:** Genes specifically enriched in red rice rhizome tissues

**SeqID**	**Length**	**Total RPKM**	**Tip**	**Zone**	**Whole rhizome**	**Root**	**Stem**	**Leaf**	**Description**
OlR_126102	686	80	38.30	2.31	37.00	2.06	0.07	0.00	Gibberellin stimulated transcript related protein 2
OlR_050984	1429	45	19.40	1.10	22.00	2.00	0.05	0.00	Growth-regulating factor 7
OlR_073115	3062	286	66.50	45.50	77.60	38.50	30.70	27.60	Oligosaccharyl transferase, putative
OlR_043253	1253	53	25.30	0.85	25.60	1.60	0.02	0.00	*O*-methyltransferase family protein
OlR_068386	291	4	1.58	0.64	1.70	0.00	0.00	0.00	*O*-methyltransferase family protein
OlR_068169	1466	904	313.00	310.70	279.30	0.24	0.35	0.19	Papain-like cysteine proteinase
OlR_036907	1707	892	351.70	256.50	282.50	0.20	0.85	0.54	Papain-like cysteine proteinase
OlR_014591	203	130	67.60	17.00	45.00	0.00	0.00	0.00	Proline-rich protein
OlR_012678	414	309	179.20	41.50	87.50	0.16	0.21	0.06	Proline-rich protein
OlR_014601	228	38	21.30	2.20	14.30	0.00	0.00	0.00	Proline-rich protein
OlR_057892	227	56	33.80	3.80	18.00	0.03	0.03	0.00	Proline-rich protein
OlR_093882	723	60	3.60	51.40	4.90	0.00	0.01	0.01	Putative uncharacterized protein
OlR_074525	1030	32	15.40	2.60	12.80	0.83	0.05	0.00	SAUR36-auxin-responsive SAUR family member
OlR_061631	816	1292	450.70	344.40	496.70	0.06	0.00	0.00	Sesquiterpene synthase
OlR_000238	449	1672	608.20	443.90	619.70	0.24	0.00	0.03	Sesquiterpene synthase
OlR_080975	468	1200	499.60	322.90	377.30	0.46	0.01	0.10	Sesquiterpene synthase
OlR_071477	2388	6	2.30	0.19	2.70	0.54	0.00	0.01	Sesquiterpene synthase
OlR_030819	1490	47	22.00	1.30	23.00	0.13	0.09	0.00	TF bHLH
OlR_019085	1240	126	54.80	14.30	55.80	0.16	1.10	0.06	TF bHLH
OlR_108847	1373	28	14.50	0.26	13.00	0.10	0.27	0.10	TF bHLH
OlR_098853	2361	47	19.90	2.10	21.80	2.70	0.01	0.01	TF Bzip
OlR_001936	1642	31	13.60	1.21	15.10	0.64	0.14	0.03	TF Bzip
OlR_142152	1280	103	31.40	44.60	26.80	0.10	0.10	0.06	TF TALE
OlR_089840	349	8	4.00	0.25	3.80	0.00	0.00	0.00	TF YABBY
OlR_002917	761	15	6.80	0.61	7.10	0.04	0.00	0.00	TF YABBY
OlR_124125	1048	738	298.00	225.70	212.70	1.60	0.10	0.04	Thionin
OlR_065478	2497	575	173.30	303.10	93.80	4.80	0.01	0.00	Thionin

Plant *O*-methyltransferases (OMTs) are known to be involved in methylation of plant specialized metabolites, especially phenylpropanoids and flavonoids that may be involved in defense against pathogens or insect pests [49]. Proline-rich proteins (PRP) are plant cell wall proteins and expression of PRPs is influenced by factors associated with biotic and abiotic stresses. However, increasing evidence suggests that the PRP proteins may have important roles in normal development and function in determining cell-type-specific wall structures during plant development, affecting cold-tolerance [50] or disease resistance [51] in rice. These genes likely play similar roles in red rice rhizome tissues. Papain-like cysteine proteases (PLCPs) play crucial roles in plant–pathogen/pest interactions [52]. Since the rhizome is an underground stem and is potentially continuously challenged by biotic (bacteria, fungi, nematodes, insects and other pathogens) and abiotic (drought, temperature and salt) stresses, the high expression of these stress and resistance-related genes in the rhizome tissue would contribute to its ability compete in its environment and perhaps may enable its invasiveness.

A total of 1,870 (3%) identified genes showed significant homology to known plant transcription factors (TFs) [53]. Transcription factors (TF) play a critical role in regulating the expression of genes involved in various physiological and developmental processes, including rhizome development. Indeed, a number of TF genes were specifically expressed or highly enriched in the rhizome (Figure 4, Table 3 and Additional file 4), especially members of the YABBY and bHLH families. Members of the YABBY family have been shown to be preferentially expressed in immature organs containing meristems and organ primordial, and this TF family appears to be involved in various functions regulating organ formation [54-56] and gibberellin metabolism [57]. Their high expression in red rice’s rhizome apical tip supports these functions. Genes in this family may therefore be very important for rhizome growth and development. The bHLH superfamily has been found to have large number of functions in essential physiological and developmental processes in plants. For example, in *Arabidopsis*, bHLH genes have been shown to be involved in regulating the differentiation of trichomes [58], and a bHLH transcription factor, UPBEAT1 (UPB1), modulates the balance between cell proliferation and differentiation by directly regulating the expression of a set of peroxidases in the root [59]. In maize, a putative bHLH transcription factor, teosinte branched1 (TB1), is involved in lateral branching and accounts partly for morphological differences in axillary branching between maize and teosinte [60]. The rice TB1 gene (OsTB1) functions as a negative regulator for lateral branching in rice [61]. Thus, the bHLH and YABBY genes expressed in red rice rhizome apical tip and elongation zone may play important roles in establishing and maintaining apical dominance and in enabling fast linear invasive growth of this species.

A putative oligosaccharyl transferase showed the highest degree of rhizome-specific expression in the rhizome producing species, Johnsongrass (*Sorghum halepense*) [13]. In our dataset, we also found an oligosaccharyl transferase gene with relatively high expression levels in rhizome tissues, but which also showed high expression levels in other tissues (root, stem and leaf) (Table 3). What role this gene plays in red rice and Johnsongrass is not clear, but it likely has a role in determining cell wall composition, and may therefore be a downstream protein compared to regulatory genes involved in differentiation and growth of rhizomes.

In contrast, we found four sesquiterpene synthase genes with high and specific expression levels in the rhizome, with three (OlR_061631, OlR_000238 and OlR_080975) of them showing extremely high expression (>1200 RPKM) in rhizome tissues including rhizome tip, zone and whole rhizome, but with very low expression (<0.5 RPKM) in root, stem and leaf (Table 3). This class of enzyme converts the isoprenoid pathway intermediate, farnesyl diphosphate, into hundreds of different sesquiterpenoids, which are important for plant defense against herbivores as well as for attraction of pollinators [62], among other functions. Some sesquiterpene synthase genes have been cloned and characterized from maize, such as *stc1 *[63] and *tps10 *[64]. Those genes produce chemical defense signals to attract natural enemies of herbivores. Furthermore, plant terpenoid formation is not only responsive to environmental (biotic and abiotic) cues, but is also related to developmental processes [65]. For example, upregulation of sesquiterpene synthase genes in *Arabidopsis* floral organs is certainly related to flower development [66]. Floral volatile terpenes also play an important role in the attraction of a variety of pollinators and thus have important implications for plant reproduction [67]. The rhizome serves as the primary energy storage and propagation/reproduction organ of rhizomatous species like red rice.

High expression of sesquiterpene synthase genes in red rice rhizome as demonstrated in this study may support the hypothesis that rhizomatous terpenoids may also play important roles in rhizome development and reproduction, perhaps acting as signaling molecules, approaching hormone function, or being major players in the rhizome chemical defense arsenal. In *Arabidopsis*, a basic helix-loop-helix (bHLH) transcription factor, MYC2, directly binds to promoters of the sesquiterpene synthase genes *TPS21* and *TPS11* and activates their expression. MYC2 is also connected to GA signaling by regulating a subset of GA-responsive genes [68]. Interestingly, in this study, we also found that a number of bHLH transcription factor genes were highly and specifically expressed in the rhizome tissue (see above), together with high expression of these sesquiterpene synthase genes (Table 3). Further investigation of the molecular mechanisms and interactions of specific members of the bHLH family, terpene synthase genes/gene products and phytohormones in rhizome tissue, the subject of future work, will provide valuable data to our understanding of rhizome development and function. Comparison to other rhizomatous species will enable us to determine whether these roles are conserved in the plant kingdom, or are specific to individual species.

### *O. longistaminata* rhizome and root proteomes

A label free GeLC-MS/MS approach was employed to identify and determine the relative abundance of proteins present in rhizome tissues (apical tip and elongation zone) and roots of *O. longistaminata*. Using this approach, we were able to identify nearly 30,000 mass spectra per biological replicate and 12,194 non-redundant peptides with a false discovery rate (FDR) of less than 1% at the protein level.

**Figure 5:**
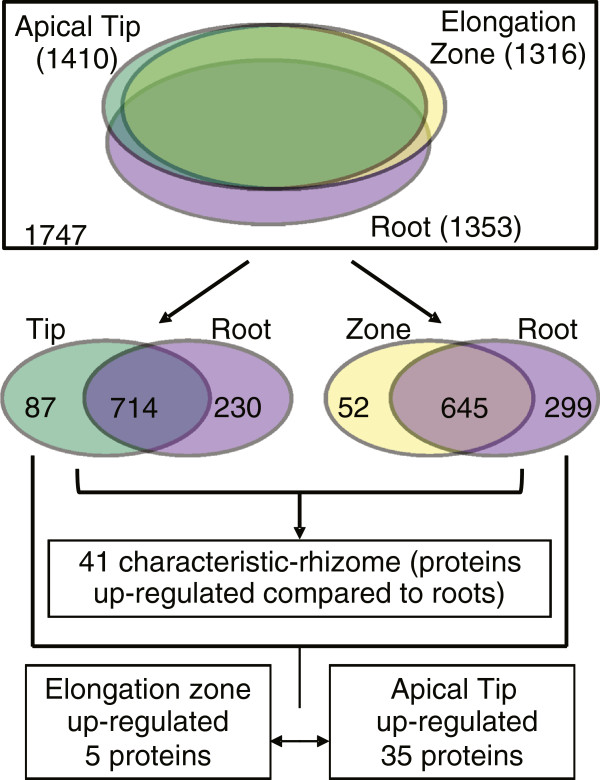
**Workflow for identification of red rice rhizome-characteristic proteins and differential regulation between the apical tip and the elongation zone proteins.** Proportional venn diagram representing the total number of proteins identified in each *Oryza longistaminata* rhizome tissues before and after spectral count quantitative analysis. A total of 1747 proteins were identified and considered reproducible (detected in at least 3 biological replicates) in the rhizome apical tip, rhizome elongation zone and roots tissues. From this amount, 74, 15 and 288 proteins were exclusively detected in the tip, zone and root tissues, respectively. Differential expression was determined using the TFold test at *p* value of 0.05. Identifications that satisfied both fold and statistical criteria were considered differently regulated. After statistical analysis, we obtained 87 and 52 proteins up-regulated in tip and zone compared to roots, respectively. Proteins found to be up-regulated in both apical tip and elongation zone were combined to create a non-redundant list of the up-regulated, characteristic proteins. Differences between tip and zone were determined after removal of those detected in the same or lower expression level than in the roots.

In total, 2,921 non-redundant protein groups (Additional file 6) were identified among the 5 biological replicates. Only proteins detected in at least 3 biological replicates were considered for downstream analysis of differentially regulated proteins between rhizomes (tip and zone) and root tissues. A total of 1,747 proteins were reproducibly detected among the biological replicates. The highest number of proteins identified was found in the tip (1,410) followed by roots (1,353) and finally the elongation zone (1,316) (Figure 5).

Among the 2,921 non-redundant proteins, 207 were of fungal origin. Interestingly, 123 (59%) of these were from *Magnaporthe oryzae*, with some being highly expressed in rhizome apical tip and/or rhizome elongation zone (see Additional file 7). For example, an ATP-dependent RNA helicase, FAL1 (OlR_047156), was detected at much higher levels in rhizome tissues than in root, especially in the rhizome tip. Other fungal proteins up-regulated in rhizome tissues included GTP-binding nuclear protein GSP1, 60S ribosomal protein L12, small COPII coat GTPase SAR1 and T-complex protein 1 subunit alpha (Additional file 7). The other 84 fungal proteins were from the genera *Neurospora* (17 proteins), *Colletotrichum* (6 proteins), *Podospora* (6 proteins), *Chaetomium* (5 proteins) and *Thielavia* (5 proteins), etc. This result was consistent with the transcriptome analyses where many *M. oryzae* unigenes were detected, especially in the rhizome tissues (see above), and confirmed the presence of this fungus within the red rice tissues.

**Figure 6:**
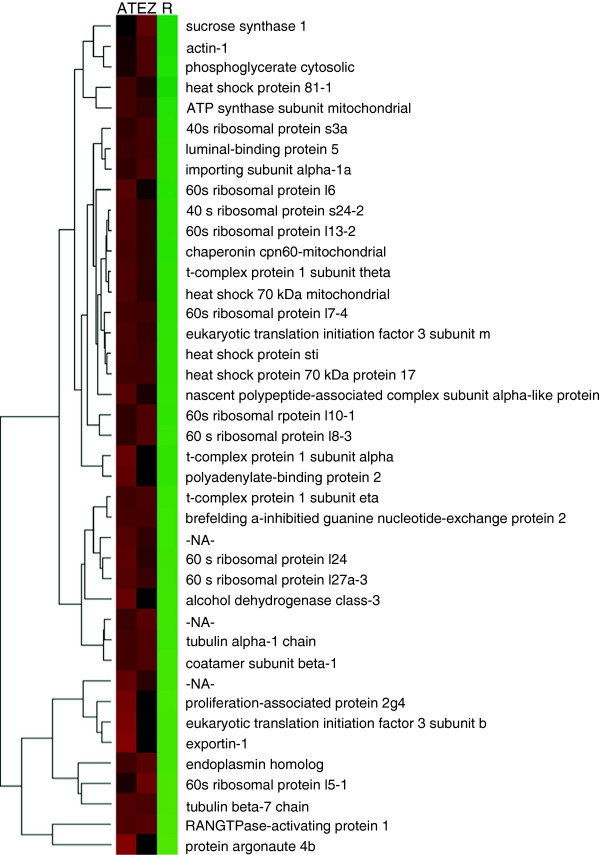
Hierarchical clustering for the 41 rhizome-characteristic proteins (AT: apical tip; EZ: elongation zone; R: root).

To detect rhizome-characteristic proteins (proteins up-regulated in both tip and zone compared to roots), pairwise comparisons and GO analysis were carried out: tip versus roots and zone versus roots. From this analysis, we detected 41 up-regulated rhizome proteins termed here as rhizome-characteristic proteins (Figure 6 and Additional file 8). To determine differences within the rhizome tissues (tip and zone), the up-regulated proteins identified in the apical tip and elongation zone compared to roots were pairwise compared, thereby detecting proteins highly enriched in only one rhizome tissue in relation to roots, and not included in the previously list of 41 rhizome characteristic proteins. A total of 40 proteins showed differential regulation between rhizome tissues (Figure 5 and Additional file 9).

**Figure 7:**
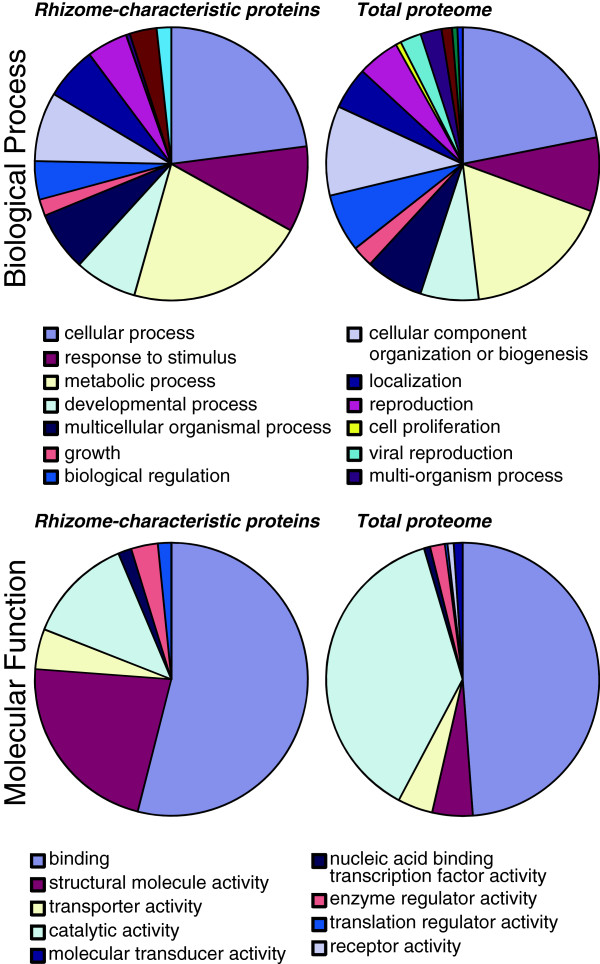
Distribution of GO biological process and molecular function terms mapped for red rice rhizome proteins.

In addition, we mapped the red rice rhizome protein distributions within the GO categories, biological process and molecular function (Figure 7). The results of this analysis showed that in the biological process category, the subcategories “cellular process” was the most abundant, followed by “metabolic process” and “response to stimulus”, and in the molecular function category, the subcategory “binding” was the most abundant, followed by “catalytic activity” and “structural molecule activity” (Figure 7). Considering the actively growing state of the tissues used in this comparison, these results are not surprising.

The proteomic results were generally consistent with the transcriptomic results, especially for those unigenes/proteins that have high expression levels in rhizome tissues (Additional file 10), such as sucrose synthase 1 (OlR_068383), tubulin alpha-3 chain (OlR_005131), heat shock protein (OlR_074205), translationally-controlled tumor protein homolog (OlR_123383) and glyceraldehyde-3-phosphate dehydrogenase (OlR_135779). However, for some highly expressed unigenes, no associated peptides/proteins could be detected, such as for cytochrome P450 like TBP (OlR_135247) and senescence-associated protein (OlR_101485). This could be explained by both biological processes and technical limitations inherent to the detection methods and the nature of the proteins in question [69]. A similar result was found for some genes examined in previous work with glandular trichomes [70,71] and developing seed [72]. Our previous work with common reed rhizome also suggested that steady state transcript and protein levels are often not proportional.

Detecting those proteins that are not concordant with transcript levels helps to reveal examples of post-transcriptional regulation. Indeed, we detected some proteins in the certain tissues whose transcript levels were not consistent with protein expression levels. There is a growing body of evidence that although in many cases protein levels are determined by transcriptional control, in other cases mRNA transcription profiles are poor predictors of protein levels because of numerous post-transcriptional regulatory activities and post-translational events that generate a high diversity of proteins [73]. In some cases, protein abundances are more conserved than mRNA abundances [74]. As a consequence, it is likely that the two complementary approaches (analysis of the transcriptome and of the proteome) used in the present work would provide a more comprehensive picture of the mechanisms that determine and control rhizomatousness in species like *O. longistaminata*.

Ten rhizome-characteristic proteins were 40S and 60S ribosomal proteins, which presented similar expression levels in apical tip and elongation zone (Additional file 8). Another six ribosomal proteins were up-regulated in tip compared to elongation zone (Additional file 9). These proteins are related to cell differentiation in meristematic tissues [75] and recently were also found to be enriched in the apical tip of *Equisentum* rhizomes [76]. An orthologous protein to the human proliferation-associated protein 2 g4 (Q9UQ80, Additional file 8), involved in ribosome biogenesis and organ growth in Arabidopsis [77], was also detected in higher levels in rhizome tissues, especially within the apical tip. Another protein up-regulated in rhizome tissues was the T-complex protein 1 (TCP1). Three accessions were found for this protein, each comprising a different subunit (alpha, theta and eta; Additional file 8), and were more highly expressed in tip compared to the elongation zone. TCP1 participates in the folding of newly translated proteins in the cytosol, including tubulins and actins [78,79]. Consistent with this finding, an actin-1 and two tubulins (alpha and beta chains) were also found to be enriched at similar levels in both rhizome tissues. Actins and tubulins have been reported to accumulate in meristematic tissues [80] and are involved in cell elongation [81,82]. In addition, a villin-3 protein, which is involved with the regulation of actin filament formation and stability [83], was up-regulated in the elongation zone compared to tip, reinforcing the idea that proteins related to the cytoskeleton may have an important role during rhizome growth and development. Proteins up-regulated in rhizome tip compared to the elongation zone (Additional file 9) were mainly related to protein fate (heat shock proteins, chaperone, 26S proteasome), protein synthesis (ribosomal proteins, elongation factors, translation factor) and RNA processing (transcription elongation factor, ribonuclease, peptidyl-prolyl cis-trans isomerase, arginine-tRNA), which would be expected due to the high level of cell division and transcription in this tissue. Thus, the proteomics data paint a picture where proteins involved in cell division, differentiation and growth are actively expressed in the rhizome apical tip and elongation zone and support the transcriptomic results.

### Red rice metabolite profiling

**Figure 8:**
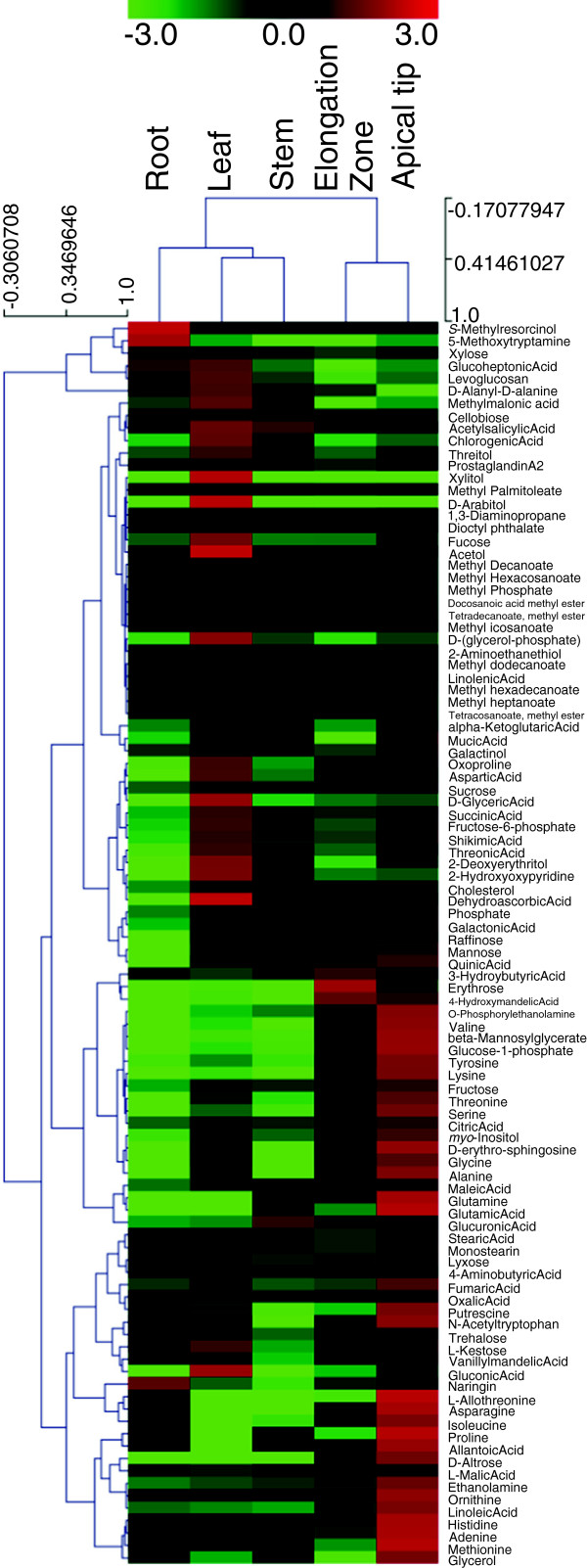
**Hierarchical clustering analysis of 100 primary metabolites in red rice tissues.** 100 primary metabolites from red rice rhizome apical tip, rhizome elongation zone, stem, leaf and root tissues, including 24 sugars, 32 organic acids, 20 amino acids and others were detected.

Using a standard metabolite profiling approach, with GC-TOFMS identification and quantification, we detected and quantified 100 primary metabolites from red rice rhizome apical tip, rhizome elongation zone, stem, leaf and root tissues, including 24 sugars, 32 organic acids, 20 amino acids and others (Figure 8, Additional file 11). As shown in Figure 8, all tissues were unique in their overall metabolite profiles, even the two rhizome tissues, the apical tip and the elongation zone. In general, the rhizome tissues had higher levels of sugars, on average, than the leaf, stem and root. Fructose and sucrose were the first and second most abundant sugars in the rhizome tip and zone, followed by glucose-1-phosphate in the tip and mannose in the zone (see Table 4). These were the most abundant sugars in the other tissues as well. Compared with the other tissues (leaf, stem and root), however, the concentrations of three sugars (D-altrose, erythrose and glucose-1-phosphate) were more than 10-fold higher in the rhizome tissues. Interestingly, although glycerol was not particularly abundant in the rhizome tissues, it was found at 24.6-fold higher levels in the tip than in the rhizome zone (Additional file 11). These results suggest that critical metabolites involved in cell division and expansion are targeted to the apical tip and elongation zone.

**Table 4:** Relative levels in all tissues of the most abundant compounds detected in red rice rhizome tissues

**Category**	**Name**	**Leaf**		**Stem**		**Root**		**Rhizome zone**		**Rhizome tip**	
		**Relative intensity**	**±SE**	**Relative intensity**	**±SE**	**Relative intensity**	**±SE**	**Relative intensity**	**±SE**	**Relative intensity**	**±SE**
Sugar	D-altrose	1.00	1.00	23.75	18.10	4.98	1.31	1366.02	823.61	2372.64	1093.74
Sugar	Erythrose	4.70	0.90	3.66	1.41	1.00	0.12	134.09	82.96	20.18	10.67
Sugar	Glucose-1-phosphate	3.74	0.55	2.61	0.94	1.00	0.29	11.85	2.95	70.13	15.38
Organic acids	4-hydroxymandelic acid	2.42	1.00	1.00	0.20	1.05	0.16	43.92	3.62	30.49	7.69
Organic acids	Allantoic acid	1.00	0.64	n.d.		n.d.		7.98	5.06	34.11	15.55
Amino acid	Asparagine	12.73	7.35	1.00	0.23	n.d.		559.05	178.00	3652.17	523.34
Amino acid	Glutamic acid	45.19	29.00	n.d.		1.00	1.00	659.09	226.01	9270.69	1185.72
Amino acid	Glutamine	5.83	2.64	n.d.		1.00	0.45	566.25	15.75	3178.44	273.06
Amino acid	Histidine	n.d.		n.d.		n.d.		1.00	0.21	5.96	0.53
Amino acid	L-allothreonine	5.57	1.63	1.00	0.65	n.d.		17.67	3.28	568.86	158.12
Amino acid	Methionine	n.d.		n.d.		n.d.		1.00	0.28	14.61	2.28
Amino acid	N-acetyltryptophan	n.d.		1.00	0.47	n.d.		11.26	1.25	32.54	2.09
Amino acid	Ornithine	n.d.		n.d.		n.d.		1.00	0.43	3.04	0.26
Amino acid	Proline	1.00	0.74	n.d.		n.d.		4.47	2.16	99.02	23.00

Note: n.d. = not detected. Relative Intensity = relative compound intensity normalized to the lowest value per compound. SE = standard error.

Among 32 organic acids, L-malate, citrate, and quinate were the most abundant (Table 4), especially in the rhizome tissues. In addition, the concentrations of 4-hydroxymandelate and allantoate were more than 15-fold higher in the rhizome tissues than in other tissues. A previous study suggested that allantoate and allantoin are the principal forms of nitrogen transported from nodulated roots to shoots of the soybean plant and the site of allantoin synthesis is the nodule [84]. More experiments will be needed to determine what role these two compounds play in the red rice rhizome, but the current evidence suggests a role in supplying much needed nitrogen for growth and differentiation of the rhizome growing tip, especially to support amino acid biosynthesis. Red rice does not possess nodules, raising the question of where these compounds are produced in this species.

**Figure 9:**
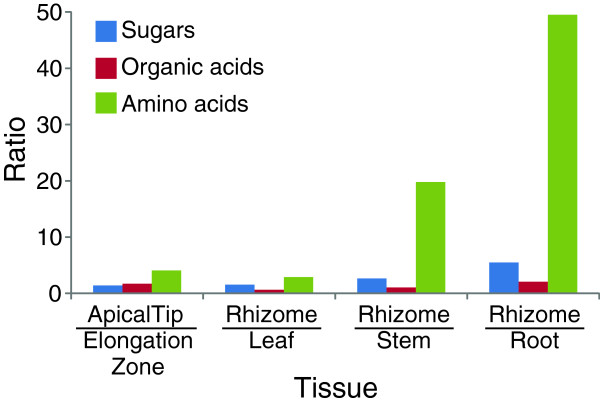
**Comparative analysis of primary metabolites in different tissues of red rice.** Amino acids were most abundant in rhizome tissue, especially in the tip compared to the other tissues.

Rhizome tissue, especially the rhizome apical tip, contained the highest levels of amino acids compared to the other tissues (Figure 9). For example, the concentrations of nine amino acids (asparagine, glutamine, histidine, glutamate, proline, N-acetyltryptophan, L-allothreonine, ornithine and methionine; see Table 4) were observed to be at least 20-fold higher compared to the root, leaf, and stem. In the rhizome tissues, asparagine, glutamine and oxoproline were the top 3 most abundant amino acids. Three amino acids (L-allothreonine, proline and glutamate) were more highly enriched in the rhizome apical tip than in the rhizome elongation zone (Additional file 11). The roles of the non-protein amino acids are unknown in this tissue.

These results could indicate a significant metabolic difference between rhizomes and other tissues, especially the rhizome apical tip, which contains the apical meristematic region. This tissue contained significantly higher levels of many important primary metabolites compared to other tissues including root, leaf and stem. This result may be an indication of the elevated metabolic demand involved with cell division and growth that occurs in this region. It is also possible that some of the large observed differences in absolute quantification of many primary metabolites may be due to the smaller average cell size of the rhizome apical tip and elongation zone compared to the more mature tissues analyzed from the leaf, root and stem, which contain much larger vacuoles as well as fiber cells. However, cell size difference cannot explain the differences in relative abundances between different compounds and classes of compounds within these various tissues, supporting the hypothesis that metabolic processes involved with rhizome growth and differentiation are significantly different from processes in the other tissues evaluated. These results further support the hypothesis that rhizomes are very different from roots, not only in anatomy (stelar structure) and gene expression, but also metabolically. The fourteen compounds from the three major classes described above as the most abundant in the rhizome tissues (see Table 4 and Additional file 11) may be particularly important for rhizome development.

As discussed above, a number of plant hormone-related genes were identified through transcriptome sequencing. We also explored the distribution of twelve plant hormones/growth regulators in the different tissues (see Table 5), including gibberellins (GA), abscisic acid (ABA), *trans*-Zeatin, *cis*-Zeatin, isopentenyl adenine (ip), indole-3-acetic acid (IAA), salicylic acid (SA), jasmonate (JA), D-SA, D-ip, D-*trans*-Zeatin and D-ABA. Four of these compounds, ABA, IAA, JA and SA, were present in all tissues, whereas the others were either not detected or present at such low concentrations that they could not be quantified in the tissue sample sizes that were available for this analysis. SA was very abundant in leaf and stem, which makes sense since those are the sites where it would be expected to be synthesized. On the other hand, ABA was most abundant in the rhizome tissues, being highest in the apical tip, which supports the model of growth regulator control of rhizome development that has been described [43]. The concentrations of three hormones (ABA, JA and SA) in rhizome tip were higher than in the zone. In general, ABA mediates changes within the vertical stem apical meristem and inflorescences, causing bud and seed dormancy. ABA is also an essential signal for plant resistance to pathogens, affecting JA biosynthesis [85]. Its role in rhizome apical meristems remains to be elucidated, but based on the fact that the tissue used for analysis was undergoing rapid growth, it appears to play a role in rhizomes that is distinct from that played in upright stems.

**Table 5:** Quantification of plant hormones detected in red rice extracts

	**ABA**	**IAA**	**JA**	**SA**
Rhizome apical tip	10.5 ± 1.9	0.038 ± 0.017	61.7 ± 22.6	389 ± 116
Rhizome elongation zone	4.18 ± 0.57	0.058 ± 0.015	45.1 ± 16.7	85.5 ± 36.4
Root	2.16 ± 0.50	0.064 ± 0.018	17.4 ± 2.0	72.2 ± 17.8
Stem	3.90 ± 0.86	0.137 ± 0.036	3.3 ± 1.2	3085 ± 680
Leaf	8.32 ± 1.72	0.119 ± 0.046	11.3 ± 2.2	6265 ± 1255

Note: Units are in pmol/g fresh weight.

## Conclusions

The ability to comprehensively probe the transcriptome, proteome and metabolome of non-model plant species led to discovery of a number of genes and proteins that are specifically expressed in the rhizome tissues of red rice, *Oryza longistaminata*. Among these were transcription factors and hormone metabolism and stress response-related genes, which likely play roles in regulating rhizome differentiation and growth. A very interesting finding was the discovery of a large set of *M. oryzae* (fungal) genes that were preferentially expressed in the rhizome, even though these plants did not show symptoms of rice blast disease. The interaction of these genes with red rice genes likely leads to the ability of this species to be resistant to the disease. And also we found evidence for the presence of a number of endophytic fungal species in red rice, especially in the rhizome tissues. Endophytes have been used as sources of antimicrobials and other biologically active specialized metabolites with pharmacological and agrochemical applications, and they are important components of plant microbiomes [30]. Because of new improvements to sequencing technologies, investigations of plant microbiomes has been carried out in *Arabidopsis* roots [86,87], although the studies were mainly focused on host-specific bacterial endophyte communities. Rhizomes and roots are underground organs and have similar soil/rhizosphere environments. More detailed investigation of the microbiomes of rhizomatous species, in particular fungal endophytes in rhizome tissue, will likely lead to great advances in our understanding of plant symbiosis [88], growth, defense, stress tolerance, invasiveness and productivity. Finally, metabolite profiling experiments further demonstrated that the rhizome tissues of red rice are significantly different from other tissues, especially the root, supporting the hypothesis that rhizomes are indeed more like stems than roots and that the rhizomes of different species likely are analogous plant organs.

## Methods

### Plant material, sample collection and histological analysis

Red rice (*O. longistaminata*) plants, originally obtained from the International Rice Research Institute, were grown in 3 gallon pots and clonally propagated in the greenhouse of the Institute of Biological Chemistry, Washington State University (Figure 1a). At the active tillering stage, rhizome apical tips (containing the meristematic region) and the neighboring elongation zones (Figure 1b) were quickly dissected from freshly dug rhizomes and flash frozen in liquid N_2_. Other plant tissues, including leaf, stem, root and whole rhizome, were similarly collected from the same plants at this time. The sampled tissues were stored at -80°C until further processed. A total of five replicate plants (five independent biological replicates for each type of tissue) were sampled. The same exact tissue samples (from a common grind) were used for RNA and protein isolation for all procedures to ensure that direct comparisons between RNA and protein data could be made. For histological analysis, rhizome nodes and internodes (Figure 1c) were cut (~1 mm) and fixed in a phosphate buffer, pH 7.0, at 4°C. Sections were stained with Toluidine blue for 10s and observed under a Nikon 4500 digital camera.

### Illumina library construction and sequencing

Total RNA and mRNA were obtained from various tissues (100 mg) and used for Illumina sequencing library construction as described previously [89].

For rhizome apical tip and elongation zone samples, we generated paired-end libraries for sequencing on different Illumina sequencing platforms: 2 × 54 bp format on GAII and 2 × 100 bp on HiSeq 2000. For leaf, stem, root and whole rhizome tissue samples, 5 individual replicate libraries for each tissue type were produced and then pooled for multiplexed sequencing on a single Illumina flowcell lane for each tissue type using the 50 bp single read format according to the manufacturer’s instructions.

Base-calling and quality value calculations, various quality controls including removal of reads containing primer/adaptor sequences, trimming of read length and filtering of high-quality reads based on the score value were performed by the Illumina data processing pipeline.

### Isolation, culture, cytological analysis and molecular manipulations of *Magnaporthe oryzae*

Fresh and healthy red rice rhizomes were collected from the greenhouse and surface sterilized as described [90]. The isolation, growth and maintenance of M. *oryzae*, media composition, and nucleic acid extraction were all performed as previously described [90,91]. For light microscopy inspection, rhizome tissues were cut (~20 μm) and fixed in a phosphate buffer, pH 7.0, at 4°C. Sections were stained with Toluidine blue for 10 s and observed under a Nikon 4500 digital camera. For scanning electron microscopy (SEM), fresh rhizome samples were processed and observed following the reported method [92].

The quality and quantity of RNA samples from hyphyae/mycelia were assessed using a NanoDrop 2000 Spectrophotometer (Thermo Scientific, Wilmington, DE), with A_260_: A_280_ ratio greater than 2.0 being deemed suitable for RNA quality. For the RT-PCR analysis, 1 μg of total RNA treated by 2 μl of RNase-free DNase (1 U/μl; Promega Inc., Fitchburg WI, USA) was reverse-transcribed using the SuperScript® II reverse transcriptase kit (Invitrogen, Carlsbad, CA, USA). The forward and reverse primers used were designed to amplify an alcohol oxidase gene that is specific to *M. oryzae*; forward primer: 5′- ATGATGACTTCCAGGCCAAG -3′ and reverse primer: 5′- AAGCGATGGGGTACGTGTAG -3′), with an expected PCR amplicon of 156 bp. The PCR reactions were run using the following reaction conditions with a final volume of 25 μl: (1) 10 mM primers (R + F); (2) 20 ng genomic DNA or 200 ng cDNA; (3) 10× buffer; (4) 0.5 U Taq. PCR was carried out with the following program: 94°C for 5 min for one cycle; 94°C for 50 s, 55°C for 50 s, and 72°C for 80 s for 34 cycles; 72°C for 10 min for one cycle. RT-PCR products were analyzed by gel electrophoresis on 1.5% agarose gels.

### Data assembly, annotation and DE (differential expression) analysis

The filtered reads were assembled using the CLC Genomics Workbench 5.0 with default settings including the scaffolding option. Potential poly-A tails were removed using EMBOSS trimest [93] followed by finalizing with MIRA and CAP3 in the iAssembler package [94]. The entire dataset has been deposited in GenBank’s Short Read Archive (SRA) under accession numbers: PRJNA196977, SRR828682, SRR830212, SRR830652, SRR833549, SRR831108, SRR834501, SRR834502 and SRR831166. It can be accessed at: http://www.ncbi.nlm.nih.gov/bioproject/?term=PRJNA196977.

The unigenes were annotated and queried using the Transcriptome Computational Workbench (TCW) [95], which is an extension of the PAVE (Program for Assembling and Viewing ESTs) v3 software [96]. Using the singleTCW manager, the assembled unigenes (contigs and singletons) were annotated with a subset of the UniProt taxonomic databases [97] and putative transcription factors (TF) from PlantTFDB [53], with assignments being made for matches that met the BLAST E-value cut-off of 1E-10. The read counts were entered into the TCW OlR database and the RPKM (reads per kilobase per million reads) values computed. The unigene DE p-values were computed with edgeR [41] and the GO p-values were computed with GOseq [42].

### Protein extraction and SDS-PAGE

Tissues from rhizome apical tip and elongation zone and root were frozen in liquid nitrogen and ground in a mortar to obtain a fine powder. Aliquots of 200 mg of the powder were submitted to phenol protein extraction as described by Balbuena et al. [76]. Protein concentration was determined using the BCA Protein Kit (Thermo Fisher Scientific, Houston, TX) using BSA as standard. Protein extracts were prepared in five biological replicates. Gel electrophoresis was performed under denaturing conditions in 12% polyacrylamide gels using 20 mA per gel. Gels were stained with colloidal Coomassie blue stain under standard conditions.

### In-gel trypsin digestion and LC-MS-MS analyses

Before protein digestion, gel lanes for each biological replicate were sliced into 10 equal size segments, diced into approximately 1 mm cubes with a clean scalpel and transferred into a 96 well plate device (MultiScreen Solvinert Plates, Millipore). Tryptic digestion was carried out according to Shevchenko et al. [98]. Each trypsin-digested and dried sample was reconstituted in 0.1% (v/v) formic acid and analyzed by nanospray-liquid chromatography – tandem mass spectrometry (nESI-LC-MS/MS) performed with a ProteomeX LTQ mass spectrometer (Thermo Fisher, San Jose) as described previously [76].

### Database searching

Sequences obtained from red rice Illumina transcriptome assembly (OlR) were translated and the open reading frames (ORFs) scanned using Virtual Ribosome software version 1.1 [99]. For each sequence, the longest ORF were reported. The protein sequences were combined to randomized sequences (i.e., decoy) that were generated using an in house developed program (DecoyDB Creator, available at http://www.oilseedproteomics.missouri.edu). Acquired MS/MS spectra were searched using SEQUEST under Bioworks 3.3.1 software package (Thermo Fisher) against the concatenated protein database (26,894 entries) and peptide-spectrum matches (PSM) validated using the Search Engine Processor tool [100]. Spectrum, peptide and protein cutoffs were adjusted to achieve a false discovery rate of 1% at the protein level for each biological replicate as described previously [76].

### Relative protein quantification

Spectral counts were used to estimate individual protein amounts in each complex protein sample. Proteins containing common peptides were grouped and relative quantification was performed considering the number of spectral counts per protein group. In this way, shared spectra were counted only once within each protein group, avoiding inadequate quantitative values. PatternLab for Proteomics [101] was used to evaluate quantitative differences between the two rhizome tissues. Spectral counts were first normalized using the Row Sigma Normalization [101] found in the PatternLab browser. Pairwise comparisons were then performed using the TFold test module [102] to detect differentially accumulating proteins between tissues. Proteins that were detected in at least three biological replicates were considered for TFold tests with a BH-q value set to 0.05. Also, the F-stringency parameter was optimized in order to maximize low abundance protein detection [102].

### Functional annotation and classification

Differently regulated proteins were annotated and functionally classified based on gene Ontology (GO) terms using the Blast2GO tool [103]. Alignments were obtained using the BLASTP program against the NCBI nr database using default parameters and 1×10^-6^ for expected value. Mapping and annotation steps were also performed using BLAST2GO default values. In addition, GO terms from the Plant GO Slim (http://www.geneontology.org/GO.slims.shtml) ontologies were assigned to each entry using the Blast2GO module. GO combined charts related to biological process and molecular function were represented at the second level.

### Hierarchical clustering

The spectral data of differentially regulated proteins between rhizome apical tip and elongation zone were clustered using Euclidean distances as a distance metric and UPGMA in the PermutMatrix software [104]. Raw spectral data were divided by the average spectral count of each clustered protein, followed by log2 transformation.

### Extraction and derivatization of primary metabolites

Leaf, stem, root, rhizome apical tip and rhizome elongation zone tissues of red rice (six biological replicates per tissue from different plants) were frozen in liquid nitrogen when collected from plants grown as described above. All samples were stored at -80°C until required for GC-MS analysis. Primary metabolites were extracted as follows. Samples were lyophilized and then disrupted in a microfuge tube using a single 5 mm steel ball and 1.0 mL extraction solvent of methanol:isopropanol:water (5:2:2) and vortexing. After 2 min centrifugation at 16,100 × g, the supernatants were collected and concentrated to dryness. The dried extracts were reconstituted with a secondary extraction solvent (acetonitrile: water (1:1)). The supernatants were dried for further analysis followed by 5 min centrifugation at 16,100 × g. 5 μl of a solution of 20 mg · ml^-1^ of 98% pure methoxyamine hydrochloride (Sigma, St. Louis, MO) in pyridine was added and shaken at 30°C for 90 min to protect aldehyde and ketone groups. 45 μl of MSTFA with 1% TMCS (1 ml bottles, Pierce, Rockford IL) was added and shaken at 37°C for 30 min for trimethylsilylation of acidic groups.

### Extraction and derivatization of primary metabolites

Leaf, stem, root, rhizome apical tip and rhizome elongation zone tissues of red rice (six biological replicates per tissue from different plants) were frozen in liquid nitrogen when collected from plants grown as described above. All samples were stored at -80°C until required for GC-MS analysis. Primary metabolites were extracted as follows. Samples were lyophilized and then disrupted in a microfuge tube using a single 5 mm steel ball and 1.0 mL extraction solvent of methanol:isopropanol:water (5:2:2) and vortexing. After 2 min centrifugation at 16,100 × g, the supernatants were collected and concentrated to dryness. The dried extracts were reconstituted with a secondary extraction solvent (acetonitrile: water (1:1)). The supernatants were dried for further analysis followed by 5 min centrifugation at 16,100 × g. 5 μl of a solution of 20 mg · ml^-1^ of 98% pure methoxyamine hydrochloride (Sigma, St. Louis, MO) in pyridine was added and shaken at 30°C for 90 min to protect aldehyde and ketone groups. 45 μl of MSTFA with 1% TMCS (1 ml bottles, Pierce, Rockford IL) was added and shaken at 37°C for 30 min for trimethylsilylation of acidic groups.

### GC-TOF MS analysis of primary metabolites

Primary metabolites were separated, identified and quantified on a Pegasus 4D GC-TOF mass spectrometry system (LECO, St. Joseph, MI) equipped with an MPS-2 Prepstation sample robot (Gerstel, Muehlheim, Germany) and an RTX®-5Sil MS with Integra-Guard® column (30 m × 0.25 mm ID × 0.25 μm film thickness) from Restek (GmbH, Bad Homburg, Germany). The oven temperature was held initially at 50°C for 1.0 min, raised at 20°C · min^-1^ to 330°C, and held for 5.0 min. The column flow (He, constant) was 1 mL · min^-1^. A sample volume of 1.0 μL was injected in splitless mode. Mass spectra were acquired from *m/z* 35–500 at a rate of 17 spectra · s^-1^. Primary metabolite data analysis was performed using ChromaTOF software version 4.41, which supports automatic deconvolution of all mass spectra from a chromatogram, built in mass-spectral correction for co-eluting metabolites, calculation of retention indices, and identification of a suitable fragment mass-to charge ratio for selective quantification.

An in-house metabolite mass spectral library and the LECO/Fiehn Metabolomics library were used for identification of compounds. Each identified metabolite was assigned a similarity value, which is a measurement of the similarity between the collected spectrum and the library mass spectrum. Identifications were only assigned if this value was higher than 500 as a cut-off value. In the Statistical Compare feature of ChromaTOF, the processed samples were added to a sample table and assigned to their respective groups. The alignment processing method provided two parameters, for retention time (RT) and for mass spectral matching. RT match criteria took into account a maximum RT difference and a maximum number of modulation periods between peaks. For spectral matching, a mass threshold and a minimum similarity match were defined. Besides a separate signal to noise ratio for peaks not found by the initial peak finding, thresholds for analytes to be kept for statistical evaluation (minimum number of samples or minimum percent of samples in a class that contain the analyte) were defined.

### Extraction of plant hormones

Bieleski buffer (methanol:chloroform:formic acid:water (12:5:2:1)) was used as extraction solvent for plant hormone analysis. The plant material (100 mg fresh weight) was placed in 2.0 ml microcentrifuge tubes and extracted in 1.0 ml of Bieleski solvent using a TissueLyser II (Qiagen, Valencia, CA) at a frequency of 27 Hz for 3 min after adding a 5 mm diam. steel ball. The tube content was ultrasonicated for 3 min and then stirred for 10 min. After centrifugation (10 min, 15,000 rpm, 4°C) the supernatant was lyophilized. The dried extracts were each dissolved in 50 μl of mobile phase (acetonitrile:water (5:95), 0.1% formic acid) prior to UPLC-MS/MS analyses.

### UPLC-MS/MS analysis of plant hormones

Plant hormones were measured in a triple quadrupole mass spectrometer (Xevo TQ, Waters, Milford, MA) using a selected reaction monitoring (SRM) MS/MS program with prior chromatographic separation by UPLC (ACQUITY UPLC System, Waters) with an ACQUITY UPLC HSS T3 column (1.8 μm, 2.1 × 100 mm, Waters). Stable isotope-labeled standard compounds were purchased from OlChemim Ltd. (Olomouc, Czech Republic) and added as standards in runs at known concentrations to allow for absolute quantification of plant hormones in these samples. Plant hormones were separated at a flow rate of 0.3 ml · min^-1^ with linear gradients of solvent A (0.1% formic acid) and solvent B (0.1% formic acid in acetonitrile) set according to the following profile: 0 min, 95% A; 0.5 min, 95% A; 7.0 min, 50% A; 7.5 min, 5% A; 10 min, 5% A; 10.5 min, 95% A; 13 min, 95% A. Capillary voltage was 2.5 kV. Cone voltage (V) and collision energy (eV) in the collision cell were as follows: salicylic acid (SA), 24 V, 24 eV; indole-3-acetic acid (IAA), 18 V, 24 eV; N^6^-isopentenyl adenine (iP), 34 V, 14 eV; jasmonic acid (JA), 26 V, 10 eV; cis-zeatin (cZ), 36 V, 34 eV; trans-zeatin (tZ), 34 V, 20 eV; abscisic acid (ABA), 28 V, 12 eV; and gibberellic acid (GA3), 34 V, 26 eV. Data were processed with MassLynx™ and TargetLynx™ software (version 4.1, Waters).

## Abbreviations

DE: Differential expression; GC-TOFMS: Gas chromatography-time-of-flight-mass spectrometry; GeLC-MS/MS: Gel electrophoresis and liquid chromatography coupled to tandem mass spectrometry; GO: Gene ontology; PAVE: Program for Assembling and Viewing ESTs; PSM: Peptide-spectrum matches; RPKM: Reads per kilobase per million reads; TCW: Transcriptome Computational Workbench; TF: Transcription factor; UPLC-MS/MS: Ultra performance liquid chromatography-tandem mass spectrometry.

## Competing interests

The authors declare that they have no competing interests.

## Authors’ contributions

RH designed and preformed the experiments, analyzed the data, and wrote the manuscript. FS and TSB carried out peptide identification and proteomic data analysis. JJP collected plant hormone and metabolite data, and assisted with metabolite analysis. MJK performed the transcriptome assembly. WN, MW and CAS performed transcriptome annotation, DE analysis and database construction. JAC and GDM assisted with sequencing. JJT and CAS gave technical advice and contributed to the study design. DRG conceived the idea, designed and coordinated the study, and edited the manuscript. All authors read and approved the final manuscript.

## Supplementary Material

Additional file 1 List of the top 20 most abundant fungal unigenes in the database. Click here for file

Additional file 2 List of rhizome-specific fungal unigenes in the database. Click here for file

Additional file 3 List of root-specific fungal unigenes in the database. Click here for file

Additional file 4 List of rhizome-enriched unigenes in the database. Click here for file

Additional file 5 List of hormone-related unigenes in the database. Click here for file

Additional file 6 List of proteins identified by GeLC-MS/MS in rhizome tissues (apical tip and elongation zone) and roots of red rice. Click here for file

Additional file 7 **Proteins from *Magnaporthe oryzae *identified in red rice tissues.** Click here for file

Additional file 8 List of 41 up regulated proteins in apical tip and elongation zone compared to roots. Click here for file

Additional file 9 List of proteins differentially regulated between the rhizome tissues of red rice. Click here for file

Additional file 10 The most abundant unigenes and proteins in the database. Click here for file

Additional file 11 Levels of three main kinds of primary metabolites in different tissues of red rice. Click here for file
